# Confinement of Triple-Enzyme-Involved Antioxidant Cascade in Two-Dimensional Nanostructure

**DOI:** 10.1021/acsmaterialslett.2c00580

**Published:** 2023-01-18

**Authors:** Adel Szerlauth, Árpád Varga, Tamara Madácsy, Dániel Sebők, Sahra Bashiri, Mariusz Skwarczynski, Istvan Toth, József Maléth, Istvan Szilagyi

**Affiliations:** † MTA-SZTE Lendület Biocolloids Research Group, Interdisciplinary Excellence Centre, University of Szeged, H-6720 Szeged, Hungary; ‡ MTA-SZTE Lendület Epithelial Cell Signaling and Secretion Research Group, Interdisciplinary Excellence Centre, University of Szeged, H-6720 Szeged, Hungary; § Department of Applied and Environmental Chemistry, University of Szeged, H-6720 Szeged, Hungary; ∥ School of Chemistry and Molecular Biosciences, University of Queensland, QLD-4072 St. Lucia, Australia

## Abstract

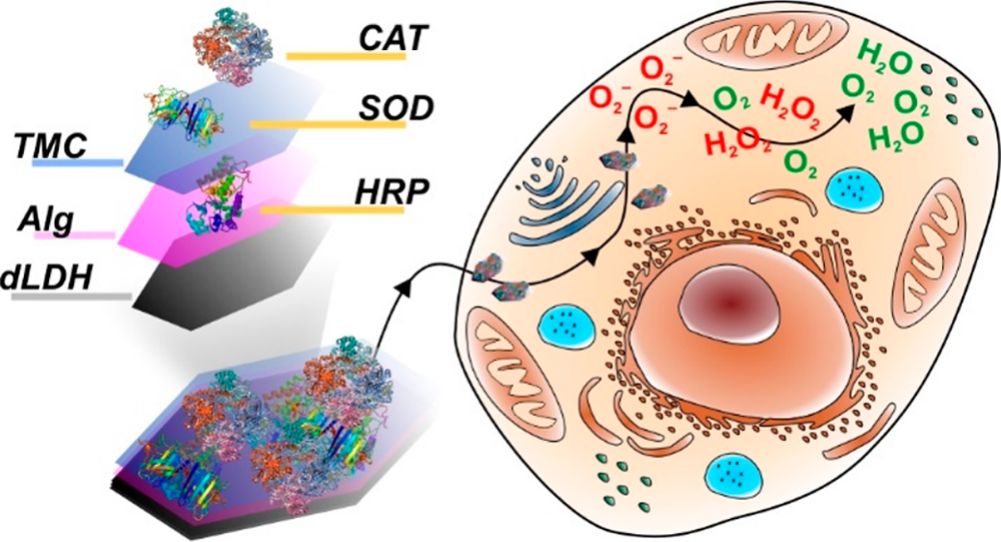

Application of antioxidant enzymes in medical or industrial processes is limited due to their high sensitivity to environmental conditions. Incorporation of such enzymes in nanostructures provides a promising route to obtain highly efficient and robust biocatalytic system to scavenge reactive oxygen species (ROS). Here, this question was addressed by confinement of superoxide dismutase (SOD), horseradish peroxidase (HRP), and catalase (CAT) enzymes into nanostructures containing polyelectrolyte building blocks (alginate (Alg) and trimethyl chitosan (TMC)) and delaminated layered double hydroxide (dLDH) nanoparticle support. The nanocomposite possessed excellent structural and colloidal stability, while antioxidant tests revealed that the enzymes remained active upon immobilization and the developed composite greatly reduced intracellular oxidative stress in two-dimensional cell cultures. Moreover, it effectively prevented hydrogen peroxide-induced double stranded DNA breaks, which is a common consequence of oxidative stress. The results provide important tools to design complex nanostructures with multienzymatic antioxidant activities for ROS scavenging.

Reactive oxygen species (ROS) such as superoxide radical anions (O_2_^–^), hydroxyl radical, or hydrogen peroxide (H_2_O_2_) play an important role in the intracellular signaling processes. However, unbalanced production of ROS, as seen in many diseases caused by toxic agents, or enhanced ROS generation by polymorphonuclear neutrophils in inflammatory processes lead to mitochondrial damage and cell death.^1−3^ In eukaryotic cells, ROS are traditionally produced during mitochondrial respiration or as the result of the activity of various enzymes (e.g., NADPH or xanthine oxidase). External stimuli such as irradiation, smoking, or the metabolism of different pharmaceutical agents may also contribute to increases in the intracellular ROS concentration. As chemically highly reactive molecules with short lifetime, ROS can damage proteins and lipids leading to oxidative stress.^4,5^ ROS are also mediators of DNA damages such as double-stranded breaks (DSBs) or 8-oxo guanine formation leading to G-T/G-A transversions.^6,7^ Beside oxidative stress, the overproduction of reactive nitrogen species (RNS) leads to nitrosative stress.^8^ For instance, the rapid reaction between nitric oxide and O_2_^–^ produces highly toxic peroxynitrite anions, which can cause DNA fragmentation or lipid oxidation, and may affect the structure of enzymes, resulting in a loss in their activity.^9^

Since elevated rates of ROS have been detected during development of cancer and inflammatory diseases, many studies aimed to restore balance between ROS and antioxidants by applying natural or synthetic ROS scavenging agents.^10−13^ However, the applicability of these methods is severely limited by their dose-dependent antioxidant capacity and the fact that they target a single point in the cascade of ROS generation.

Ideally, ROS are balanced with molecular and enzymatic antioxidants,^14^ which prevent oxidative stress by stabilizing or deactivating free radicals.^15−17^ The two most important enzymatic antioxidants present in mammalian cells are superoxide dismutase (SOD) and catalase (CAT), which neutralize O_2_^–^ and H_2_O_2_ molecules, respectively.^18^ Since H_2_O_2_ is also produced during dismutation of O_2_^–^ by SOD, these enzymes are involved in cascade reactions. In addition, horseradish peroxidase (HRP) of similar function as CAT is one of the most efficient antioxidant enzymes in plants.^19^ Furthermore, molecular antioxidants have also important role in the decomposition of ROS, both endogenous (e.g., glutathione) and exogenous (e.g., vitamins, carotenoids, and polyphenols) representatives exist.^20^

Although enzymatic antioxidants show the greatest efficiency against oxidative stress, their sensitivity to the environmental conditions (e.g., temperature, ionic strength, and pH) prevents their widespread application in antioxidant therapies.^21^ To address this challenge, immobilization of such enzymes attracted widespread contemporary interest in the scientific and technological communities.^22−24^ Apart from attachment of a single enzyme to suitable supports, joint confinement of dual enzyme cascades in hybrid materials was also the focus of several research groups.^25−27^ For example, SOD and HRP were successfully immobilized on the surface of layered double hydroxide (LDH) nanoparticles by the sequential adsorption method involving polyelectrolytes as building blocks.^28^ In another work, SOD and chloroperoxidase were coanchored on monodisperse iron oxide particles leading to successful inhibition of tumor proliferation.^26^

As described above, immobilization of antioxidant enzyme cascades provides a promising way to develop efficient and robust ROS scavenging agents for therapy or prevention of oxidative stress, in which bare enzymes fail due to their high sensitivity. It is also expected that confinement of different enzymes in composites may lead to similar cascade systems and cooperativity as in the intracellular environment. In the present work, immobilization of three antioxidant enzymes (SOD, CAT, and HRP) on 2-dimensional (2D) delaminated LDH (dLDH) nanosheets using polyelectrolytes (alginate (Alg) and trimethyl chitosan (TMC)) in the sequential adsorption method^29,30^ is described. Note that this is the first study that reports on the development of triple-enzyme-involved antioxidant nanocomposites.

As per the nanoparticulate support, LDHs are known as brucite-type anionic clays of positive structural charge, which is compensated with interlayer anions.^31,32^ Beside the original lamellar structure, single-layer LDHs have attracted the attention of several researchers, since the anisotropy of such unilamellar nanosheets allows basic investigations and modeling of 2D quantum dots to be applied as building blocks in hybrid materials as well as for delivery of various biomolecules.^33−35^ Besides, Alg and TMC were chosen to optimize charge balance and enhance enzyme adsorption during composite preparation, since these polyelectrolytes have been proven previously as suitable compounds in preparation of biocompatible nanohybrids.^36−38^ The structural, functional, and colloidal stability of the system was fully characterized by scattering, microscopy, and spectroscopy techniques, while the antioxidant activity was assessed in biochemical tests and by advanced microscopy techniques in cellular experiments.

The preparation of the 2D particles is detailed in the Supporting Information (SI), and X-ray diffraction (XRD) measurements were performed to prove their formation (Figure S1A). As a result of the delaminated morphology, no characteristic reflection bands could be detected in the 5°–50° 2θ range, indicating that the material contains thin nanosheets instead of the lamellar LDH structure.^39^ However, the reflections at ∼61° 2θ (belonging to Miller indices (110) and (113)) are proofs for the formation of 2D dLDH particles.^40,41^

Atomic force microscopy (AFM)-based height profile assessment (a typical measurement is shown in Figure S1B in the SI) further proved the delaminated structure of the particles. The average thickness of the dLDH particles was determined to be 1.75 ± 0.25 nm meaning that one particle consists of 1–2 layers. Transmission electron microscopy (TEM) images (inset of Figure S1A in the SI) indicate disk-like thin dLDH sheets, with an average thickness of (1.75 ± 0.35) nm in excellent agreement with data from the AFM experiments.

Small-angle X-ray scattering (SAXS) measurements were performed in both dispersion (EMBL BioSAXS synchrotron) and solid state (benchtop SAXS device) to unambiguously show the difference between single layer nanosheets and the classic lamellar LDH structure. Plotting the scattered intensities (*I*(*h*)) as a function of the scattering vector (*h*) in different representations ([*h*^3^*I*(*h*)] vs *h*^3^, or [*h*^4^*I*(*h*)] vs *h*^4^, for line or point collimation, respectively), the differences in the scattering curves unambiguously confirm the 2D morphology for dLDH. Accordingly, in an ideal two-phase structure with sharply defined phase boundaries (e.g., smooth surface of an individual lamella), the scattering intensity has an asymptotic feature (Figure S1C in the SI), while, due to electron density fluctuation or stacked structure, positive deviation from the asymptotic behavior can be observed (Figure S1D in the SI).^42−44^ Interpreting the results with the Porod law (eq S6 in the SI), a slope equal to 4 at higher scattering angles (*h* > 0.5 nm^–1^) clearly indicates the flat interface (i.e., an individual lamella with a smooth surface), while the deviation from the power law h^–3^ refers to a nondelaminated state, in which the system appears as a quasi-surface fractal. More details on the colloidal properties of the dLDH particles can be found elsewhere.^45^

The effect of polyelectrolyte adsorption on the charge of dLDH particles was investigated in zeta potential measurements (Figure 1).

**Figure 1:**
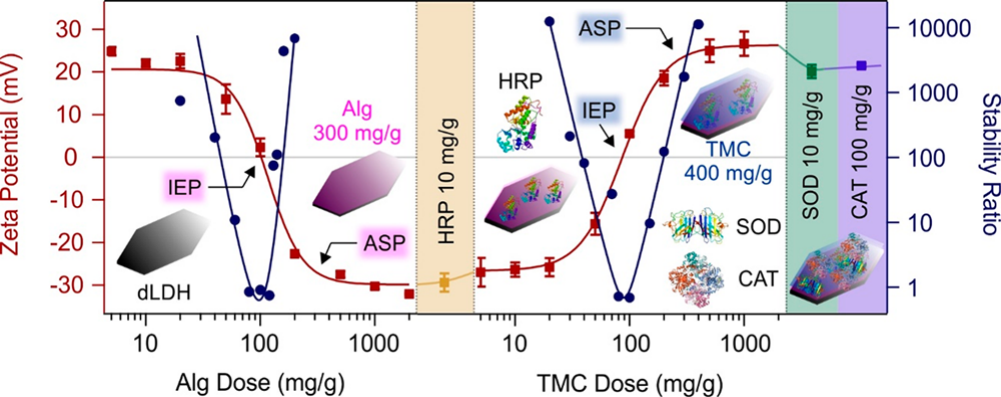
Effect of polyelectrolyte (Alg and TMC) and enzyme (HRP, SOD, and CAT) adsorption on the zeta potentials (squares, converted from the measured electrophoretic mobility data by eq S1 in the SI) and on the stability ratios (circles, determined by time-resolved DLS using eq S4 in the SI) of the particles. The mg/g unit refers to mg polyelectrolyte or enzyme adsorbed on 1 g of particles. The solid lines serve to guide the eyes.

In the first step, dLDH was functionalized with oppositely charged Alg species. The particles possessed a zeta potential value of approximately +20 mV at low Alg doses, while increasing level of the polyelectrolyte led to charge neutralization at the isoelectric point (IEP ≈ 100 mg/g) and subsequently, charge reversal due to the strong adsorption of Alg on the oppositely charged dLDH surface. A plateau observed in the zeta potentials at high doses indicated that the particles are fully covered with the polyelectrolytes at high doses. The onset of this plateau was found at 200 mg/g Alg concentration. Similar behavior was reported in other LDH-polyelectrolyte system earlier.^46,47^

Stability ratios (calculated via eq S4)^48,49^ were determined in time-resolved dynamic light scattering (DLS) measurements in the same Alg concentration range as in the mobility study to assess the colloidal stability of the systems. At low polyelectrolyte doses, the particles were stable due to the significant positive charge and strong repulsion between the electrical double layers. Around the IEP, the dispersions turned to be unstable and rapid particle aggregation occurred indicated by stability ratios close to unity. Above 200 mg/g, where the dLDH particles were completely coated with Alg, the dispersions were stable, as demonstrated by constant hydrodynamic radii measured at different intervals (see Figure S2 in the SI). Such a stability is due to the sufficiently high magnitude of the surface charge and subsequent presence of strong double layer interparticle repulsion. Accordingly, the stability ratio data could be described with a characteristic U-shaped curve, which was also reported in other oppositely charged particle–polyelectrolyte systems.^28,37,46,47,50^

The above charge–aggregation relation agrees well with the prediction of the classical Derjaguin, Landau, Verwey, and Overbeek (DLVO) theory, which describes the colloidal stability of particle dispersions with the superposition between repulsive double layer and attractive van der Waals forces.^51,52^ Based on these results, 300 mg/g Alg dose (dLDH/Alg) was used in further investigations, under which conditions the particles are highly charged and form a stable dispersion.

Similar trend, but with the opposite charge balance was observed for TMC adsorbing on the dLDH/Alg particles (see Figure 1, as well as Figure S2 in the SI). Stable dispersions were observed below and above the IEP, while unstable ones were observed near this dose. Based on the results of electrophoretic mobility and stability ratio measurements, a TMC dosage of 400 mg/g was chosen (dLDH/Alg/TMC) for further measurements.

Since biomedical and industrial applications of antioxidant agents usually occur in the presence of electrolytes,^16,20^ the resistance against salt-induced aggregation is a crucial issue. Therefore, the charging and aggregation properties of the bare and polyelectrolyte coated particles were compared in salt solutions of different concentrations (see Figure S3 in the SI). The magnitude of the zeta potentials progressively decreased by increasing the ionic strength due to charge screening by the salt constituent ions present in the solution. By fitting the zeta potential data with the Gouy–Chapman model (eq S2 in the SI),^53^ surface charge densities of +18, −25, and +8 mC/m^2^ were determined for dLDH, dLDH/Alg, and dLDH/Alg/TMC, respectively.

The increase in the ionic strength led to a decrease in the stability ratios giving rise to diffusion-controlled aggregation above the critical coagulation concentration (CCC, determined from the stability ratio data using values obtained from eq S5 in the SI (see Figure S3). This tendency can be further demonstrated by the time-resolved DLS data (Figure S4 in the SI), in which the slopes of the hydrodynamic radii versus time plots increase with the NaCl concentration. The CCCs were 21, 265, and 33 mM for dLDH, dLDH/Alg, and dLDH/Alg/TMC, respectively. Comparing the surface charge and CCC data, the high stability of dLDH/Alg can be explained by the strong electrical double layer forces. However, the surface charge densities predict lower CCC for dLDH/Alg/TMC than for dLDH, while the tendency is the opposite. This deviation from the DLVO theory is due to the presence of steric repulsion,^54,55^ which originates from the overlap of the adsorbed polyelectrolyte chains and subsequent rise in the osmotic pressure upon the approach of polyelectrolyte-covered particles.

To achieve enzyme immobilization, positively charged HRP^19^ was adsorbed on the dLDH/Alg surface, while SOD and CAT, as negatively charged enzymes under the conditions applied,^18^ were immobilized after TMC coating. The chosen enzyme loadings were 10, 10, and 100 mg/g for HRP, SOD and CAT, respectively, since these doses did not affect the zeta potential of the polyelectrolyte-coated dLDHs significantly (Figure 1) and thus, high colloidal stability was maintained in each step of the sequential adsorption process. Therefore, the final dLDH/Alg/HRP/TMC/SOD/CAT composite (denoted as dLDHaHtSC later) contained 300 mg/g Alg, 10 mg/g HRP, 400 mg/g TMC, 10 mg/g SOD, and 100 mg/g CAT, and this hybrid material was used in the antioxidant tests discussed later.

To reveal structural features of the composite, synchrotron SAXS measurements^56^ were performed in dispersions (see Figure 2A, as well as Figure S5 in the SI).

**Figure 2:**
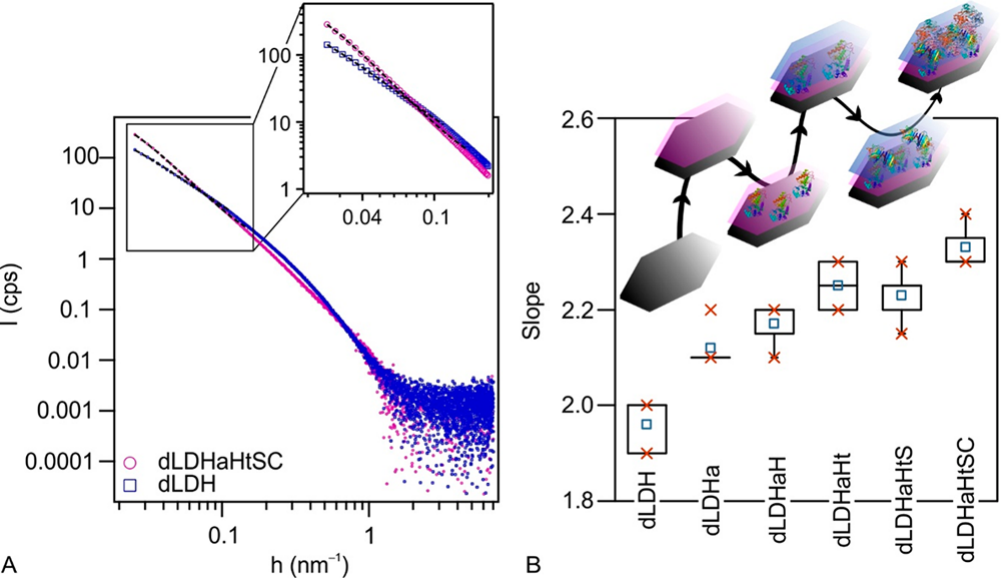
(A) Double-logarithmic plot of the scattering curves of dLDH and dLDHaHtSC and (B) the box chart representation of the slopes of the fitted lines at small angles (*h* < 0.1 nm^–1^) for the subsystems of dLDHaHtSC. The center lines represent the median, the mean is marked by empty squares in the middle of the boxes, the top and bottom “x” marks on the boxes indicate the 75% and 25%, while whiskers extend to a minimum and a maximum value. The inset in panel (B) represent the inhomogeneous distribution of the enzymes and polyelectrolytes on the surface.

The morphological and surface properties of the samples were characterized by the slope (*S*) fitted on the scattering curves at small angles (*h* < 0.1 nm^–1^) in fractal representation. For the initial dLDH sample, the power law decay of the scattering curve at small angles (*S* = 2) is characteristic for a delaminated structure with a smooth surface (Figure 2B).^42,57^ Upon formation of layers of the macromolecules in the individual steps of the sequential adsorption process, the value of the exponent gradually increased to *S* = 2.3, which indicates increasing electron density fluctuations at the interface, i.e., the surface became more diffuse once the adsorption of enzymes and polyelectrolytes occurred. Such a diffuse structure implies that the macromolecules did not adsorb in homogeneous and compact layers, but rather in a random assembly (see inset of Figure 2B) leading to a diffuse interfacial structure, which was advantageous for the antioxidant capacity, as discussed later.

Transmission electron microscopy (TEM) micrographs did not show any morphological changes after the functionalization of the dLDH material (Figure S6), and the surface-modified particles possessed shapes typical for single-layer nanosheets.

To determine the HRP, SOD, and CAT activity, biochemical test reactions were performed as follows. A guaiacol assay was used to measure the HRP activity of the bare enzyme and the dLDHaHtSC.^58^ By fitting data with the Michaelis–Menten model (eq S8 in the SI),^59^ maximum reaction rate (*v*_max_) and Michaelis constant (*K*_M_) values were calculated. The activity of bare and immobilized HRP are shown in Figure 3A, while the calculated *K*_M_ and *v*_max_ values are presented in Table S1 in the SI. It was found that both *v*_max_ and *K*_M_ values decreased upon immobilization. The latter result indicates that the enzyme has higher affinity to the substrate after heterogenization on the particle surface than in native form, which is rather surprising, compared to the literature data (Table S2 in the SI), in which an increase was often found in the *K*_M_ values after enzyme attachment to surfaces.^28,60−62^ This is due to the advantage of the heterogenization of the enzyme, since guaiacol molecules are attracted by the particle surface and, hence, are brought closer to the attached HRP molecules.

**Figure 3:**
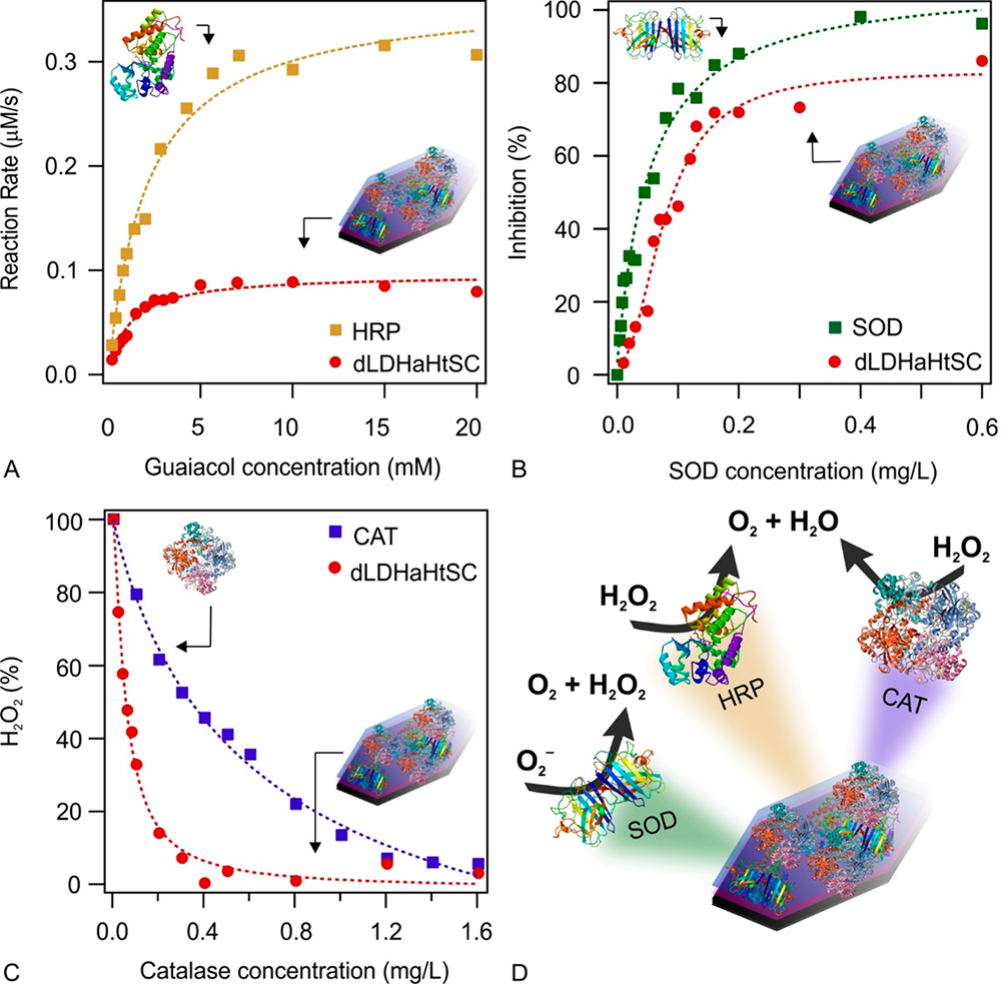
(A) Guaiacol, (B) Fridovich, and (C) CAT assays measured with the native enzymes (squares) and the dLDHaHtSC composite (circles). The dotted lines are fits used to calculate the parameters describing the enzyme-like activities reported in Table S1. (D) Scheme illustrating the enzyme-catalyzed reactions.

The Fridovich assay^63^ was applied to estimate the activity of native and immobilized SOD. Nitro blue tetrazolium was used to detect the O_2_^–^ concentration generated by the reaction between xanthine and xanthine oxidase. The inhibition (eq S7 in the SI) data, as a function of SOD concentration, is shown in Figure 3B. The maximal inhibition was ∼80%, while 0.079 mg/L IC_50_ value (enzyme dose, at which half of the forming O_2_^–^ molecules are decomposed) was observed for dLDHaHtSC meaning that it possesses very comparable activity to the native enzyme (see Table S1). This finding is consistent with the trend found in previously published IC_50_ data determined with native and immobilized SOD (Table S2).^24,28,62,64−66^

To estimate the effect of immobilization on the CAT function, a simple spectrophotometric method based on the reaction between H_2_O_2_ and ammonium molybdate was used with slight modifications.^67^ In Figure 3C, the remaining percentage of H_2_O_2_ (i.e., the fraction of the nondecomposed substrate molecules) are shown as a function of the CAT concentration. As the level of the enzyme increased, the amount of H_2_O_2_ in the test solution decreased, indicating significant CAT activity. The effective concentration (EC_50_) value, the enzyme concentration that is necessary to decompose half of the H_2_O_2_ in the reaction mixture, was significantly lower for dLDHaHtSC than for the native CAT enzyme. This result contrasts with earlier reports on CAT activity changes upon heterogenization of the enzyme (see Table S2),^24,68^ where lower enzymatic activities were determined after immobilization.

The above results point out that (i) the obtained dLDHaHtSC composite shows excellent antioxidant features and (ii) the enzymes kept their function once adsorbed on the surface, leading to the formation of a broad-spectrum ROS scavenging hybrid material.

Potential cytocompatibility of dLDHaHtSC was investigated by a microscopy-based apoptosis/necrosis detection assay, frequently used for simultaneous quantification of viable, apoptotic, and necrotic cells.^69^ Accordingly, HeLa cells were treated with dLDHaHtSC at 20 mg/L concentration for 30 min at 37 °C. In this kit, apoptosis is detected by Apopxin Green that binds to the phosphatidyl serin translocated to the outer plasma membrane during apoptosis, whereas necrosis is detected by 7-AAD, which stains the available DNA in the necrotic cells. Neither apoptotic (green staining of the cell membrane) nor late apoptotic/necrotic (red nuclear) staining were detected after dLDHaHtSC treatment, only viable cells with blue Cytocalcein Violet 450 staining were observed (Figure 4A). This was also quantified by normalizing the recorded fluorescent intensity values to the intensity of the live cells (Figure 4A).

**Figure 4:**
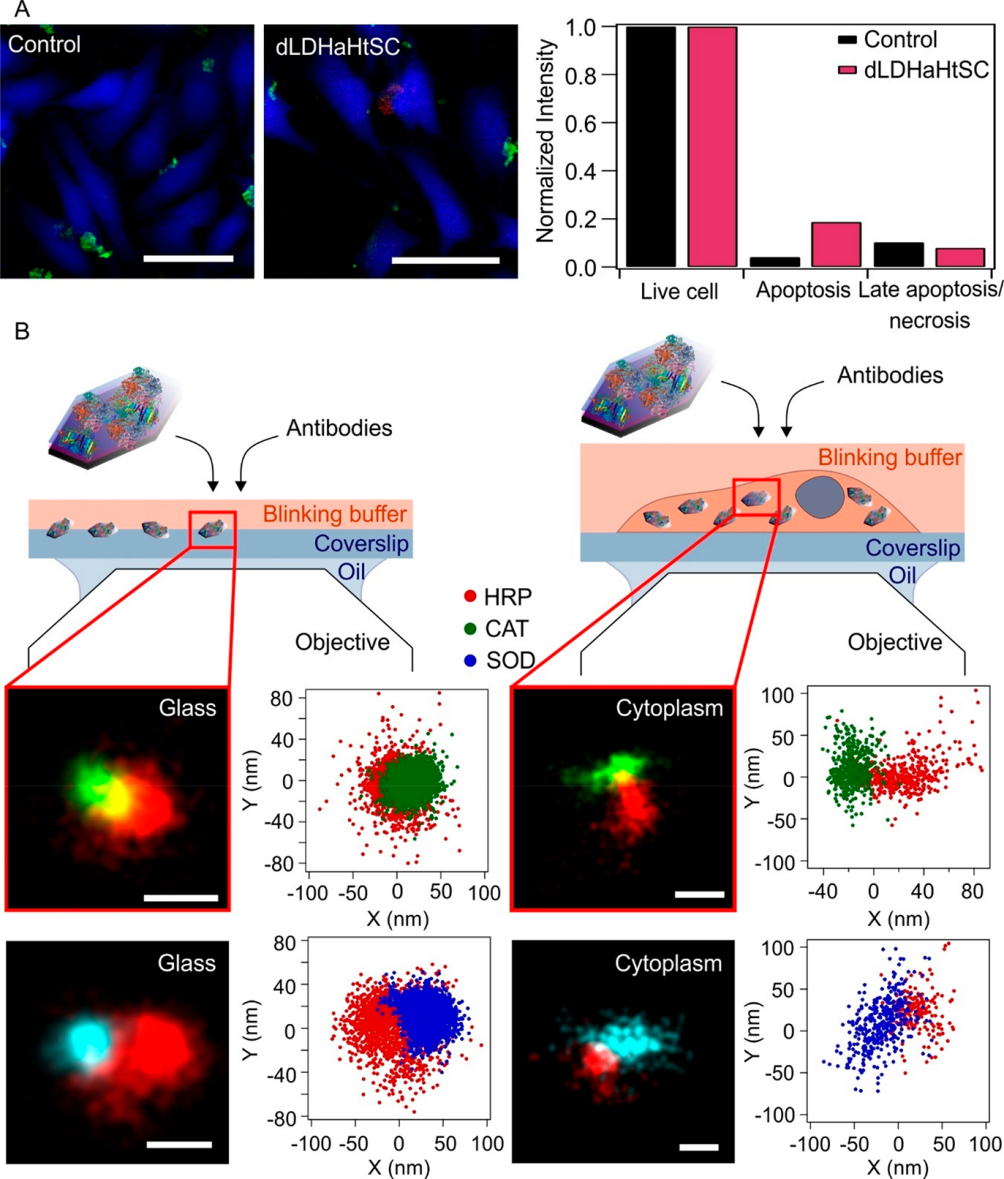
(A) Incubation with dLDHaHtSC composite did not induce apoptosis or necrosis in HeLa cells. Confocal images and normalized fluorescent intensity values demonstrate no sign of cytotoxicity of dLDHaHtSC in cell viability tests (scale bars = 50 μm). (B) Direct stochastic optical reconstruction microscopy (dSTORM) images of the fluorescently labeled particles on glass surface (left panel) and in the cytoplasm (right panel) after cellular uptake (scale bars = 50 nm). Blinking events of the fluorophores were plotted along the *X* and *Y* nanometer axes.

Thereafter, the super-resolution direct stochastic optical reconstruction microscopy (dSTORM) technique was used for size determination of the antibody-labeled dLDHaHtSC particles. Because of the technical limitations caused by the low number of commercially available antibodies, the spatial extent of dLDHaHtSC was determined by dual labeling (HRP/CAT or HRP/SOD), relative to HRP. First, the spatial extent of dLDHaHtSC was quantified on a glass surface, which revealed that the colocalizing clusters of fluorophores were ∼150–180 nm (Figure 4B). In the next step, the measurements were repeated in cellular context. Therefore, HeLa cells were incubated with 20 mg/L dLDHaHtSC for 30 min at 37 °C and dSTORM images of the perinuclear/cytoplasmic areas of the cells were captured in total internal reflection fluorescence (TIRF) mode. In this focal plane, colocalizing clusters of the two fluorophores were detected, which were identical to the clusters found on the glass surfaces (Figure 4B). These results suggest that the dLDHaHtSC particle can penetrate living cells without triggering any detectable damage. Moreover, the cellular uptake does not induce any distortion of the nanoparticles. The experimental details of the cellular experiments are described in the SI.

As discussed above, the proper balance between antioxidants and ROS is a crucial aspect to avoid oxidative stress.^1^ Therefore, the question whether our composite is able to restore this equilibrium in the presence of increased ROS production or not was addressed. To assess ROS scavenging activity, HeLa cells were loaded with 2′,7′-dichlorodihydrofluorescein diacetate (H_2_DCFDA) ROS indicator and treated with menadione, a conventional oxidative stress inducing molecule, in the presence or absence of dLDHaHtSC.^70^ As expected after the addition of menadione, ROS production was triggered rapidly in the control cells, leading to the oxidation of H_2_DCFDA and to an increase in the fluorescent intensity (Figure 5A). In contrast, this maximal fluorescence intensity change was significantly decreased (by 48.2%) in the dLDHaHtSC pretreated HeLa cells, suggesting a global intracellular ROS scavenging activity of the composite.

**Figure 5:**
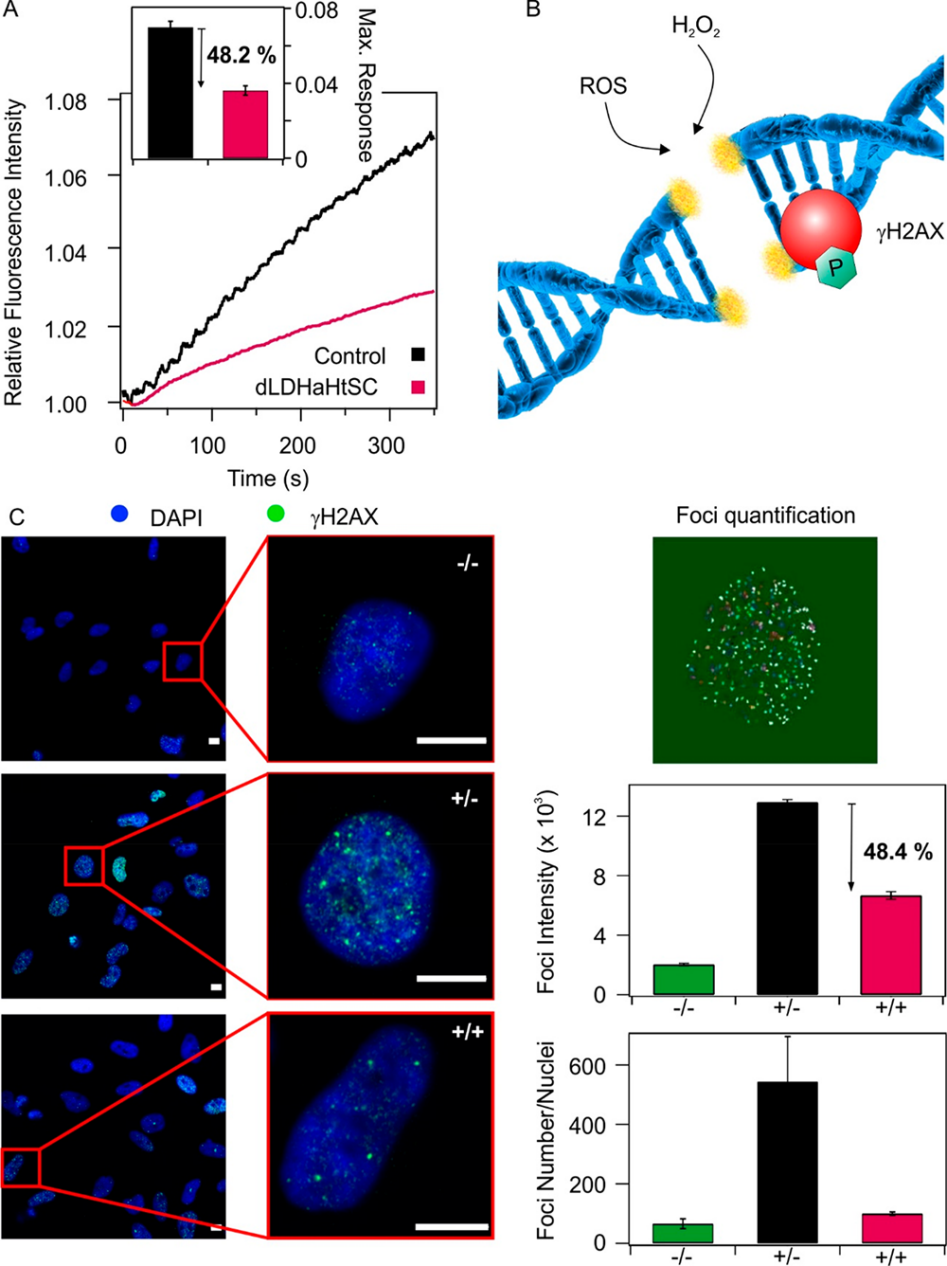
(A) ROS scavenging activity and protective effect of dLDHaHtSC against DNA damage. Significant difference in relative normalized fluorescent intensity indicates ROS production as a function of time in control and dLDHaHtSC pretreated cells. (B) Schematic illustration of the presence of γH2AX in ROS-induced DSBs. (C) DSB detection assay by γH2AX labeling were carried out to reveal the effect of dLDHaHtSC treatment versus control (scale bars = 10 μm). The absence (−) or presence (+) of H_2_O_2_ and the composite are indicated: −/– (none), ± (H_2_O_2_) and +/+ (H_2_O_2_ and dLDHaHtSC).

Next, in a proof-of-concept experiment, it was demonstrated that the developed hybrid compound is not only able to prevent unbalanced ROS production, but that this effect is sufficient to protect the cells from serious consequences of the oxidative stress. As mentioned earlier, uncontrolled production of ROS may lead to irreversible mitochondrial dysfunction or compromising genome stability through DNA damages.^71^ The H2AX histone plays a significant role in DNA damage sensing process by its phosphorylation at serine 139, γH2AX resulted in the recruitment of checkpoint factors to the DSB sites (Figure 5B).^72,73^ Therefore, the formation and quantification of γH2AX foci is a potential method for comparable ROS-induced DSB detection.^74^ Accordingly, the cells were treated with 50 μM H_2_O_2_ for 40 min (±) to induce DSB resulting in increased fluorescent intensity of the antibody-labeled γH2AX foci, compared to the control group (−/−) (Figure 5C). However, dLDHaHtSC pretreated cells (+/+) showed significantly lower fluorescent foci intensity, compared to H_2_O_2_-treated cells (48.4%), while the number of γH2AX foci was not only reduced but roughly restored to the control level. Therefore, our results suggest that the dLDHaHtSC is capable of restoring intracellular ROS-antioxidant balance under oxidative stress conditions and, thus, protects cells from global ROS overproduction and oxidative damage, such as DSB.

In summary, three antioxidant enzymes (HRP, SOD, and CAT) were successfully immobilized on single-layer dLDH nanosheets to develop a highly active broad-spectrum antioxidant composite against ROS. The polyelectrolyte dose (Alg and TMC) was optimized in light scattering measurements to tune colloidal stability and enhance enzyme immobilization during the sequential adsorption procedure. Synchrotron-based SAXS measurements revealed that polyelectrolyte and enzyme adsorption on the dLDH surface gave rise to a diffuse interface rather than compact layers. The dLDHaHtSC hybrid showed remarkable activity in both biochemical test reactions and in cellular ROS scavenging experiments. Based on the results of enzymatic assays, one can clearly conclude that the enzymes kept their biocatalytic function upon confinement in the hybrid material, as affinity of HRP to the substrate increased, SOD activity remained almost unchanged, while CAT immobilized in dLDHaHtSC showed higher H_2_O_2_ decomposing ability than the native enzyme. The presence of the enzymes in the composite was confirmed by dSTORM images before and after cellular uptake. Cell viability tests proved that the obtained hybrid material did not have any side effects on cells, since neither apoptotic nor necrotic cell death were detected in considerable amount after incubation. Pretreatment of cells with dLDHaHtSC decreased the intracellular oxidative stress significantly and prevented DNA DSBs in the presence of H_2_O_2_. The demonstrated ROS scavenging ability of the composite particle and the results of our physiological as well as live-cell experiments suggest the potential applicability of the composite in the treatment of inflammatory diseases characterized by oxidative stress.

## Supporting Information Available

Experimental details, procedures, SAXS, XRD, DLS data as well as TEM and AFM images (PDF)
