# Supplementary material for: Confinement of Triple-Enzyme-Involved Antioxidant Cascade in Two-Dimensional Nanostructure
Source: ACS Mater Lett. 2023 Jan 18;5(2):565–73. doi: 10.1021/acsmaterialslett.2c00580 (PMC9906813; doi:10.1021/acsmaterialslett.2c00580)
Supplement: Supplementary file 1 — tz2c00580_si_001.pdf [file tz2c00580_si_001.pdf]

Supporting Information (SI)

Confinement of Triple-Enzyme-Involved Antioxidant Cascade in 2-dimensional Nanostructure

Adel Szerlauth,[1‡] Árpád Varga,[2‡] Tamara Madácsy,[2] Dániel Sebők,[3] Sahra Bashiri,[4] Mariusz Skwarczynski,[4] Istvan Toth,[4] József Maléth,[2*] and Istvan Szilagyi[1*]

[1]MTA-SZTE Lendület Biocolloids Research Group, Interdisciplinary Excellence Centre, University of Szeged, H-6720 Szeged, Hungary

[2]MTA-SZTE Lendület Epithelial Cell Signaling and Secretion Research Group, Interdisciplinary Excellence Centre, University of Szeged, H-6720 Szeged, Hungary

[3]Department of Applied and Environmental Chemistry, University of Szeged, H-6720 Szeged, Hungary

[4]School of Chemistry and Molecular Biosciences, University of Queensland, QLD-4072 St Lucia, Australia

*Corresponding authors. Email: maleth.jozsef@med.u-szeged.hu (J.M.) and szistvan@chem.u-szeged.hu (I.S.)

‡These authors contributed equally to the work.

Materials

$Mg(NO_3)_2 \times 6\ H_2O$, $Al(NO_3)_3 \times 9\ H_2O$, ammonia solution (ca. 25 wt%), horseradish peroxidase (HRP), sodium alginate (Alg), sodium chloride (NaCl), sodium hydroxide (NaOH), xanthine, nitro blue tetrazolium chloride monohydrate (NBT), guaiacol, hydrogen peroxide (H_2O_2, 30 wt%), ammonium molybdate (para) tetrahydrate, disodium hydrogen phosphate anhydrous (Na_2HPO_4), and sodium dihydrogen phosphate (NaH_2PO_4) were obtained from VWR International. Catalase (CAT) from bovine liver, superoxide dismutase (SOD) from bovine liver, xanthine oxidase, 2'7'-dichlorodihydrofluorescein diacetate (H_2DCFDA), Triton-X-100, anti-HRP monoclonal antibody, glucose oxidase, glucose, and cysteamine hydrochloride were purchased from Sigma Aldrich. Dulbecco's modified eagle medium (DMEM), 10% fetal bovine serum (FBS), kanamycin sulfate, antiobiotic-antimycotic solution, GlutaMaxTM supplement, and Dulbecco's phosphate buffer saline (PBS) were obtained from Gibco. Alexa Fluor 488 Goat anti-Rabbit, Goat anti-Rabbit Alexa Fluor 568, and Donkey anti-Mouse Alexa Fluor 647 secondary antibodies were purchased from Thermo Scientific. The 4',6-diamidino-2-phenylindole (DAPI), anti-CAT, and anti-SOD1 were bought from Invitrogen. Filter set for 2',7'-bis-(2-carboxyethyl)-5-(and-6)-carboxyfluorescein acetoxymethyl ester (BCECF-AM) was obtained from Biotium, 4% paraformaldehyde from Alfa Aesar, 10% bovine serum albumin in PBS (BSA-PBS) from Pan-Biotech, primary antibody against γH2AX histone from Cell Signalling Technology, two component adhesives from Picodent, while human cervical adenocarcinoma (HeLa) cells from ATCC and the apoptosis/necrosis detection assay from Abcam. The above substances were analytical grade and used without further purification. Trimethyl chitosan (TMC) was synthesized and purified as detailed elsewhere[1]. Ultrapure water was obtained by a Puranity TU 3+ UV/UF system equipped with a UV irradiation unit (VWR International). The measurements were performed at 25 ^{o}C unless otherwise indicated.

Synthesis of layered double hydroxide nanosheets (dLDHs)

The preparation of dLDH particles was performed based on a method reported earlier[2]. In brief, a mixed $Mg(NO_3)_2$ and $Al(NO_3)_3$ salt solution was prepared, with a total salt concentration of 0.3 mol/L and a Mg/Al molar ratio of 3. The pH of the reaction system was held at ~10 by adding ammonia solution (~7 wt%) dropwise under N_2 atmosphere. After 5 minutes vigorous stirring, the mixture was treated for 30 minutes in an ultrasonic bath. The sample was washed 3-time with deionized water by centrifugation (for 10 minutes at 4200 rpm). The final stock dispersion contained homogeneously distributed dLDH particles, which was used for further measurements.

Light scattering experiments

For both electrophoretic and dynamic light scattering (DLS) measurements, a Litesizer 500 (Anton Paar) instrument was used. This device is equipped with a 40 mW laser source operating at 658 nm wavelength. During sample preparation for electrophoretic mobility measurements, calculated amount of polyelectrolyte (either Alg or TMC) and NaCl salt solutions were added to the dLDH dispersions, and the samples were equilibrated overnight. Enzymes, such as SOD, HRP and CAT, were immobilized on the composites with the same procedure as the polyelectrolytes, i.e., with simple mixing and equilibration. The total volume was 2 mL, the pH was set to 7, while the particle concentration was adjusted to 10 mg/L. The Smoluchowski equation was applied to calculate the zeta potential (ζ) values from the electrophoretic mobilities (μ)[3]:

$$\mu = \frac{\varepsilon \cdot \varepsilon_0 \cdot \zeta}{\eta} \tag{S1}$$

where ε is the relative permittivity of water, ε_0 is the permittivity of the vacuum, and η is the dynamic viscosity of the solvent. The reported zeta potential values were the average of 5 individual measurements.

To assess the surface charge density (σ) of the bare or functionalized particles, NaCl concentration was systematically varied in electrophoretic measurements and the Gouy-Chapman model was used for the calculations as follows[4]:

$$\sigma = \frac{2 \cdot k_B \cdot T \cdot \varepsilon \cdot \varepsilon_0 \cdot \kappa}{e} \sinh \frac{e \cdot \zeta}{2 \cdot k_B \cdot T} \tag{S2}$$

where k_B is the Boltzmann constant, T is the absolute temperature, κ is the inverse Debye length and e is the elementary charge.

The same sample preparation procedure was applied during DLS measurements as above, with the difference that the monitoring of the change of hydrodynamic radius (R_h) at different intervals was started right after mixing the reaction components. During salt induced aggregation measurements, NaCl concentration was systematically varied.

The R_h values were determined by applying the Cumulant fit to the intensity correlation function and then, the conversion of the translational diffusion coefficient (D) to R_h was performed by the Stokes-Einstein equation[5]:

$$R_h = \frac{k_B \cdot T}{6 \cdot \pi \cdot \eta \cdot D} \tag{S3}$$

To express the colloidal stability of the systems under different experimental conditions, stability ratio (W) was defined as[6]:

$$W = \frac{k_{fast}}{k} \tag{S4}$$

where k is the apparent aggregation rate determined from the increase of hydrodynamic radius by time in aggregating particle dispersions (see Figure S4 for some examples), while k_{fast} was measured upon diffusion-controlled particle aggregation achieved at 1 M NaCl concentration, at which repulsive forces are screened. Note that stability ratio close to one means that the dispersion is unstable and all particle collision result in formation of aggregates.

The critical coagulation concentration (CCC) values, which typically separate slow and fast aggregation regimes in particle dispersions, were calculated from the stability ratio versus NaCl concentration plots as[7]:

$$W = 1 + \left(\frac{CCC}{c_s}\right)^{-\beta} \tag{S5}$$

where c_s is the concentration of the monovalent salt, while β is the slope of the stability ratio data in the slow aggregation regime before the CCC.

Small angle X-ray scattering (SAXS)

The bare particle and the sub-systems of dLDHaHtSC were investigated at five different particle concentrations, namely, 0.2 mg/mL, 0.4 mg/mL, 0.8 mg/mL, 1.0 mg/mL, and 1.2 mg/mL with SAXS at the EMBL BioSAXS synchrotron beamline P12.[8] The scattering curve of the powder LDH sample was recorded using a bench-top SAXS device equipped with a position-sensitive detector (PSD 50M, M. Braun AG) containing 1024 channels of 55 μm in width. The CuKα radiation was generated by a Philips PW1830 X-ray generator operating at 40 kV and 30 mA. The scattering vector (h) was defined as $h=4\pi \sin\theta \lambda^{-1}$, where θ is one-half of the scattering angle and λ = 0.15406 nm is the wavelength of the CuKα radiation. The results were analyzed with the equation of Porod law[9,10]:

$$I(h) = I_0 \cdot h^{-S} \tag{S6}$$

where I is the scattered intensity and S is the slope, which describes the shape of the scattering curve at certain angles.

X-ray diffraction (XRD)

A Bruker D8 Advanced diffractometer was used for the XRD measurements in concentrated (gel-like) dispersions. The diffractometer is equipped with CuK_{α} (λ = 0.1542 nm) as a radiation source and the experiments were carried out at ambient temperature in the 5-80° 2θ range applying 0.02 step size.

Microscopy

Morphological study was carried out by atomic force (AFM) and transmission electron (TEM) microscopy. The TEM micrographs were taken by a FEI Tecnai G2 type electron microscope, while the AFM images were collected with a Multimode Nanoscope Illa AFM instrument. For TEM measurements, the dLDH dispersion and the functionalized dLDH particles were dried on carbon mesh grid and 200 kV accelerating voltage was used for imaging in the bright field mode. The AFM device was used in tapping mode in air at ambient temperature using Si tip cantilever (Veeco Nanoprobe Tips RTESPA model). The dLDH dispersion was deposited on a mica substrate (Ted Pella, Highest Grade V1), which was freshly cleaved before the measurement.

Enzyme activity tests

The Fridovich method[11] was used to determine the SOD-like activity of the materials. The final reaction mixture contained 100 μL xanthine (3 mM), 50 μL nitro blue tetrazolium (NBT, 3 mM), 150 μL xanthine oxidase (3 g/L) in phosphate buffer and it was completed to 1.5 mL with the bare enzyme or the composite samples. The enzyme concentration was systematically varied during the test reactions. After mixing the reaction components, the absorbance was monitored at 565 nm for 6 minutes. The inhibition (I) of the NBT-superoxide radical anion reaction was calculated by comparing the change in the absorbance value without any enzymatically active components (ΔA_0) and the one measured with the actual sample (ΔA_s) as:

$$I = \frac{\Delta A_s - \Delta A_0}{\Delta A_0} \qquad (S7)$$

After plotting the determined I value against the enzyme concentrations, the IC_{50} values were calculated. The IC_{50} corresponds to the enzyme concentration, which caused 50% inhibition, i.e., half of the superoxide radicals were decomposed by the catalysts.

HRP activity was determined by the guaiacol assay[12]. The total 1.8 mL reaction mixture contained 125 μL phosphate buffer (100 mM), 125 μL enzyme or composite (enzyme concentration was always 1 mg/L), 350 μL H_2O_2 (9 mM), and 1.2 mL aqueous solution of guaiacol substrate of various concentrations. The change in the absorbance data was monitored at 470 nm wavelength for 6 minutes. The reaction rate (v) was calculated from the absorbance versus time diagrams, and these v values were plotted against the substrate concentration (S). The data were fitted by the Michaelis-Menten function using the following equation[13]:

$$v = \frac{v_{max} S}{K_M + S} \tag{S8}$$

where v_{max} is the maximum reaction rate, while K_M is the Michaelis constant.

To assess CAT activity, a simple spectrophotometric assay was applied[14], with slight modification in the previously reported protocol. The total volume of the reaction mixture was set to 2.5 mL. First, 255 μL H_2O_2 (30 mM) was completed to 500 μL with CAT solution or antioxidant composite dispersion. The CAT concentration was systematically varied during the tests. After three minutes time, the reaction was stopped by the addition of 2 mL ammonium molybdate solution. The reaction between H_2O_2 and ammonium molybdate resulted in a yellow color complex, with an absorption maximum at 350 nm wavelength. Based on the measured absorbances, the remained percentage of H_2O_2 as a function of the CAT concentration was calculated, and the EC_{50} values were determined. EC_{50} is the enzyme concentration, which is necessary for the decomposition of 50% of the initial H_2O_2 in the reaction mixture.

For all enzyme activity measurements, a Thermo Fisher Genesys 10S dual beam spectrophotometer was used and the absorbances were recorded in standard plastic cuvettes (VWR). The average error for the above enzyme tests is 10%.

Cell lines

HeLa cells (ATCC-CCL-2) were grown in DMEM containing 10% FBS, 1X Kanamycin sulfate, 1X Antibiotic-Antimycotic solution and 1X GlutaMaxTM supplement. Cells were grown in a humidified incubator at 37 °C in 95% relative humidity and 5% CO_2.

Fluorescent microscopy

Intracellular concentration of reactive oxygen species (ROS) was measured as described earlier[15] by loading the cells with H_2DCFDA. The HeLa cells were plated onto cover glasses (VWR), then were preincubated for 30 minutes with 20 mg/L dLDHaHtSC composite and mounted on an Olympus IX71 fluorescent microscope equipped with an MT-20 illumination system. Filter set for BCECF-AM was described previously[16].

Apoptosis/necrosis detection assay

To detect cell death, the HeLa cells were plated onto cover glass in a 6-well plate. The cells were treated with 20 mg/L dLDHaHtSC for 30 minutes and then, were washed twice with the assay buffer followed by incubation in a reaction mixture containing 100-time diluted Apopxin Green, 200-time diluted 7-AAD, and 200-time diluted Cytocalcein 450 in assay buffer for 30 minutes at 37 °C in a humidified incubator. Next, cells were washed twice with fresh assay buffer and were visualized with a Zeiss LSM 880 confocal microscope at excitation/emission wavelengths of 490/525 nm for Apopxin Green, 550/650nm for 7-AAD, and 405/450 nm for Cytocalcein Violet 450.

ROS induced DNA damage visualization by immunofluorescent labelling

The HeLa cells were grown on cover glasses and incubated with 20 mg/L dLDHaHtSC for 30 minutes. After a washing step with 1X PBS, the cells were treated with 50 μM H_2O_2 in HeLa feeding media for 40 minutes. The cells were fixed in 4% paraformaldehyde in PBS for 15 minutes and then washed in 1X PBS for 3×5 minutes. Antigen retrieval was performed by 0.01% Triton-X-100 for 15 minutes prior to another washing step with 1X PBS for 3×5

minutes. Cells were blocked with 10% BSA-PBS for 2 h at 37 °C. After blocking, the primary antibody against γH2AX histone diluted in 1:400 ratio with 10% BSA-PBS was administered and then, left for overnight incubation at 4 °C. Samples were washed for 3×5 minutes in 1X PBS followed by use of secondary antibody (Alexa Fluor 488 Goat anti-Rabbit) in 1:800 dilution with 10% BSA-PBS and incubation for 3 h at room temperature under protection from light. Nuclear staining and mounting were carried out simultaneously with ProLong™ Gold Antifade mounting medium with DAPI and microscope slides (Fisher Scientific). Statistical image analyses were carried out by Fiji ImageJ2 (Version:2.3.0/153f) software, while number and intensity values of γH2AX focuses were calculated by Foci Picker3D plug-in.

Direct stochastic optical reconstruction microscopy (dSTORM)

The dSTORM imaging was performed by a Nanoimager S (Oxford Nanoimaging ONI Ltd) instrument. The HeLa cells were treated, fixed, permeabilized and nonspecific binding sites were blocked by the same manner as described before for γH2AX immunofluorescent labelling (see above for more experimental details). The anti-CAT and anti-SOD1 antibodies were applied in dual labelling setup together with anti-HRP monoclonal antibody to detect double positive enzyme particles. All primary antibodies were diluted in 1:200 ratio with 10% BSA-PBS and applied along with the samples for overnight incubation at 4 °C. The samples were then washed three times for 10 minutes with 1X PBS. Goat anti-Rabbit Alexa Fluor 568 and Donkey anti-Mouse Alexa Fluor 647 secondary antibodies were used in 1:400 dilution with 10% BSA-PBS. After another washing step with sterile 1X PBS (3×10 minutes), the cover glasses were placed on cavity slides (Sigma) filled with blinking buffer and sealed with two-component adhesive. Blinking buffer contained 100 U glucose oxidase, 2000 U CAT, 55.56 mM glucose, and 100 mM cysteamine hydrochloride in 1 mL final volume with sterile PBS.

Statistical analysis of cellular measurements

Statistical analysis was performed with Graphpad Prism software. All data are expressed as ± standard error of the mean. Both parametric and nonparametric tests were used based on the normality of data distribution. The P value below 0.05 was considered statistically significant. Strength of significance was determined by GraphPad Prism software.

Table S1. Enzymatic activity parameters determined for native and immobilized enzymes.

**Sample**	**v_{max} (μM/s)**	**K_M (mM)**	**IC_{50} (mg/L)**	**EC_{50} (mg/L)**
HRP	0.370 ± 0.010	2.12 ± 0.24	—	—
SOD	—	—	0.053 ± 0.007	—
CAT	—	—	—	0.660 ± 0.230
dLDHaHtSC	0.096 ± 0.004	1.13 ± 0.15	0.079 ± 0.007	0.056 ± 0.005

Table S2. Activity of SOD, HRP and CAT enzymes before and after immobilization.

Host material	Enzyme	Change in activity	Reference
Halloysite nanotubes	SOD	IC_{50_SOD}: 0.04 mg/L<br>$IC_{50\_immobilized\ SOD}$: 0.02 mg/L	17
Titania nanosheets	SOD	IC_{50_SOD}: 0.069 mg/L<br>$IC_{50\_immobilized\ SOD}$: 0.057 mg/L	18
Mg/Al-CO_3 LDH	SOD	IC_{50_SOD}: 0.069 mg/L<br>$IC_{50\_immobilized\ SOD}$: 0.056 mg/L	19
Mg/Al-CO_3 LDH	HRP	v_{max}^{HRP} = 2.8 mM/s<br>$v_{max}^{immobilized\ HRP}$ = 2.72 mM/s<br>K_M^{HRP} = 3.23 mM<br>$K_M^{immobilized\ HRP}$ = 5.25 mM	20
Titania nanosheet	HRP	comparable activity (pH 7.3)<br>(no exact v_{max} and K_M values are given)	21
Sulphate latex (SL)	papain (PPN) and HRP	$v_{max}^{PPN\ and\ HRP}$ = 0.125 mM/s<br>$v_{max}^{SL-PPN-HEP-HRP}$ = 0.069 mM/s<br>$K_M^{PPN\ and\ HRP}$ = 3.52 mM<br>$K_M^{SL-PPN-HEP-HRP}$ = 29.16 mM	22
Mg/Al-Cl LDH	SOD and HRP	IC_{50_SOD}: 0.069 mg/L<br>$IC_{50\_immobilized\ SOD}$: 0.078 mg/L<br>v_{max}^{HRP} = 0.0046 mM/s<br>$v_{max}^{immobilized\ HRP}$ = 0.038 mM/s<br>K_M^{HRP} = 1.56 mM<br>$K_M^{immobilized\ HRP}$ = 2.64 mM	15
Titania nanosheets	SOD and HRP	IC_{50_SOD}: 0.1 mg/L<br>v_{max}^{HRP} = 0.59 mM/s	23

(TNS)		$IC_{50\_immobilized\ SOD}$: 0.22 mg/L 1.30 mg/L 0.15 mg/L 0.38 mg/L	$v_{max}^{immobilized\ HRP} =$ 0.1 mM/s 0.41 mM/s 0.31 mM/s 0.34 mM/s K_M^{HRP} = 3.41 mM $K_M^{immobilized\ HRP} =$ 23.50 mM 13.13 mM 3.91 mM 15.50 mM	
		The different IC_{50}, v_{max} and K_M values for immobilized SOD and HRP is due to the different order of adsorbed polyelectrolyte and enzyme layers.		
Polydopamine/ polyethyleneimine/ titania microcapsules	CAT	v_{max}^{CAT} = 28.57 mM/min $v_{max}^{immobilized\ CAT}$ = 3.07 mM/min K_M^{CAT} = 45.02 mM $K_M^{immobilized\ CAT}$ = 49.64 mM		24
Hollow silica nanospheres	SOD and CAT	relative activity of SOD: 100% relative activity of immobilized SOD: 18.6% relative activity of CAT: 100% relative activity of immobilized CAT: 62.7%		25

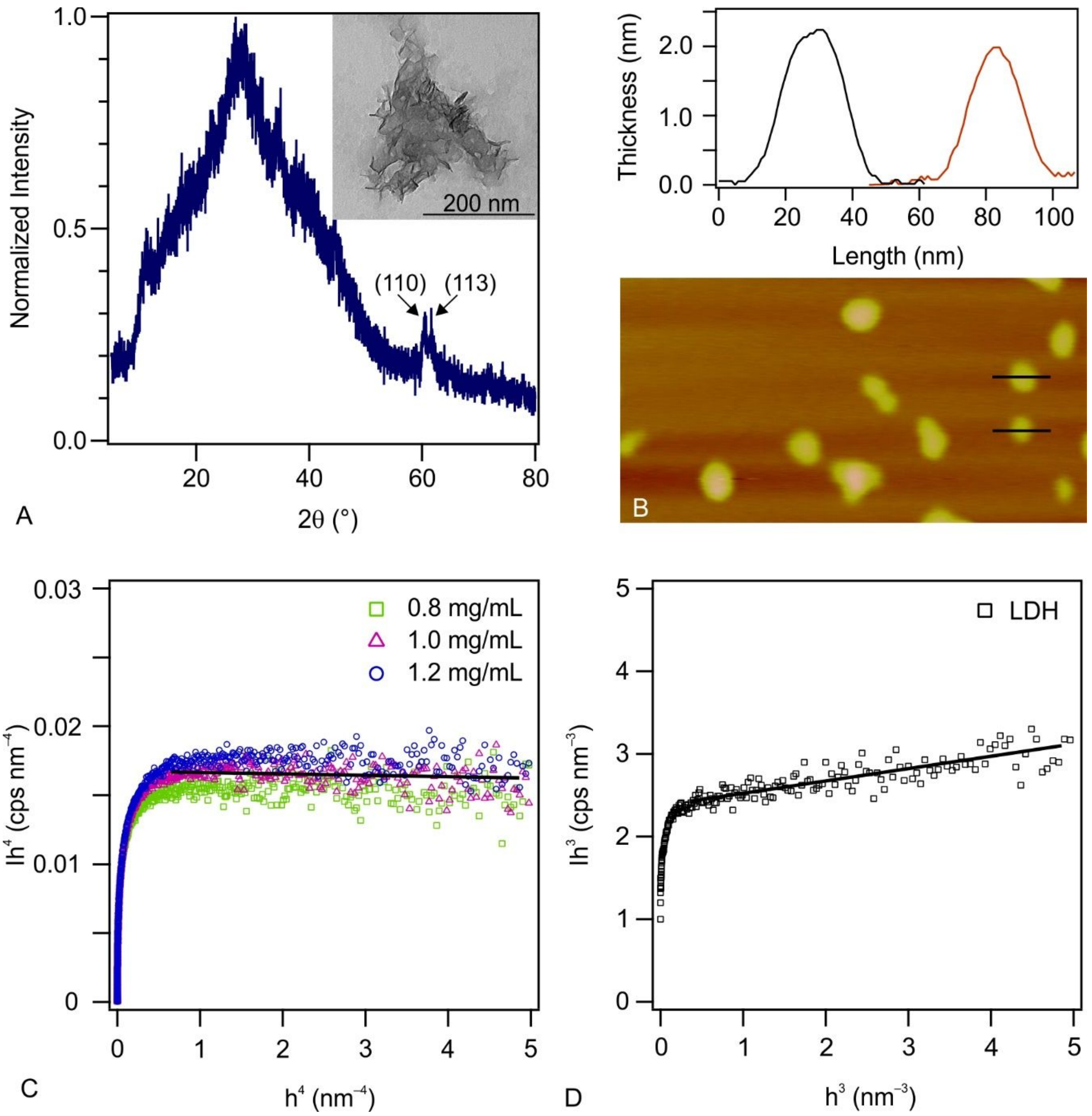

Figure S1. XRD diffractogram (A) and AFM image with height profile (B) of dLDH particles. The inset in (A) shows a typical TEM image of the particles. The SAXS curves of dLDH at different particle concentrations in dispersions (C) and in powder form (D) in Porod representation (the power exponents are 4 and 3 for point-collimation and line-collimation, respectively).

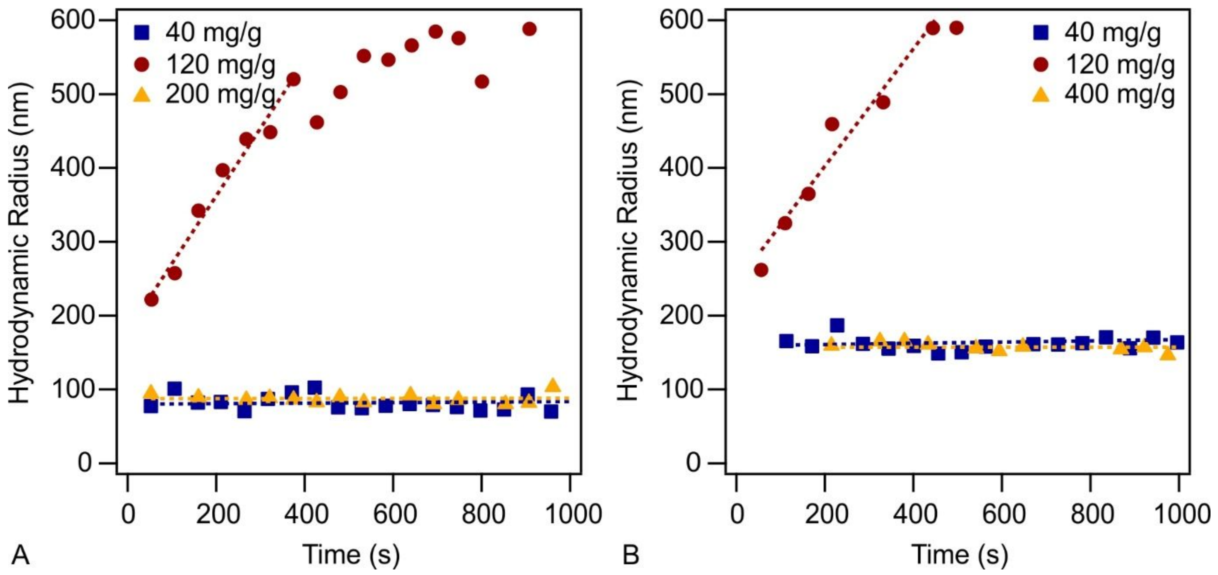

Figure S2. Change in the hydrodynamic radii as a function of time at different Alg (A) and TMC (B) doses. The mg/g unit refers to mg polyelectrolyte or enzyme adsorbed on 1 g of particles. The solid lines were used to calculate the apparent aggregation rates and subsequently, the stability ratios with equation S4.

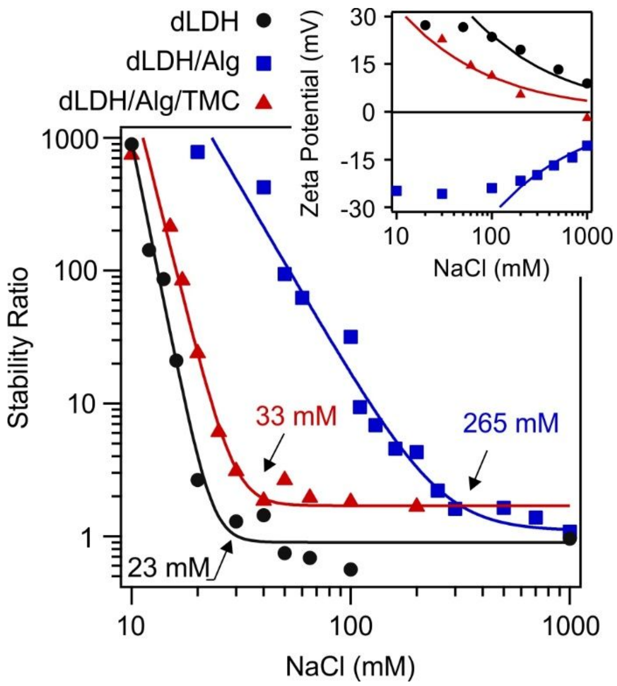

Figure S3. Stability ratio of bare and polyelectrolyte coated dLDH particles measured at different NaCl concentrations. The inset shows the zeta potential values as a function of NaCl concentration. The average measurement error for stability ratios and zeta potentials are 10% and 5 mV, respectively. The solid line in the inset were calculated with equation S2 and with equation S5 for the stability ratios. The CCC values are indicated with arrows.

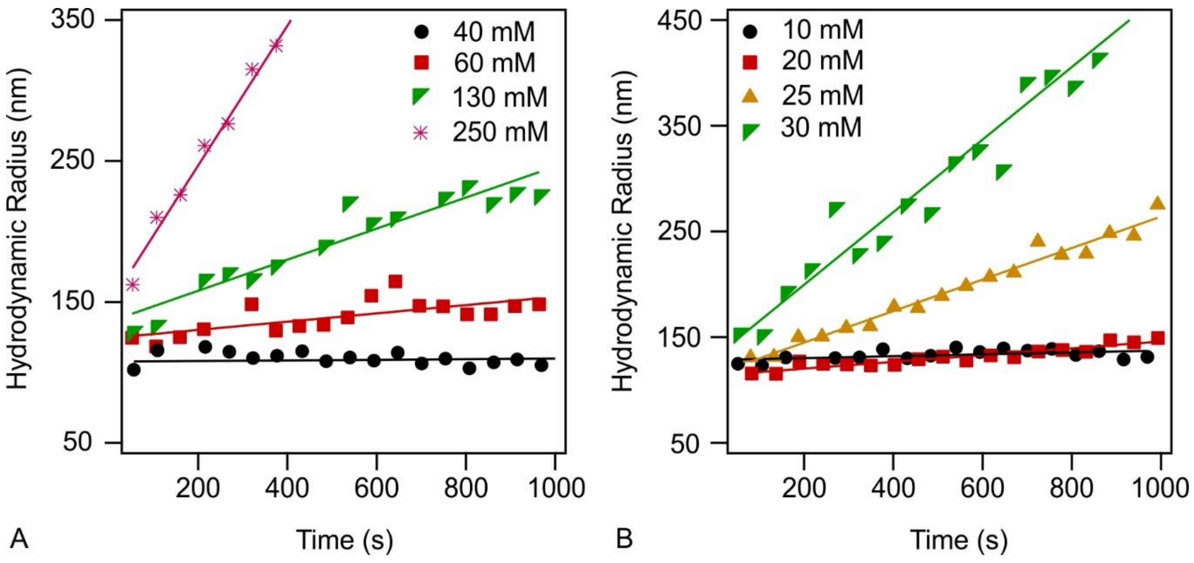

Figure S4. Hydrodynamic radii versus time graphs for dLDH/Alg (A) and dLDH/Alg/TMC (B) particles at different NaCl concentrations. The fitted lines were used to calculate the apparent aggregation rates and the stability ratios with equation S4.

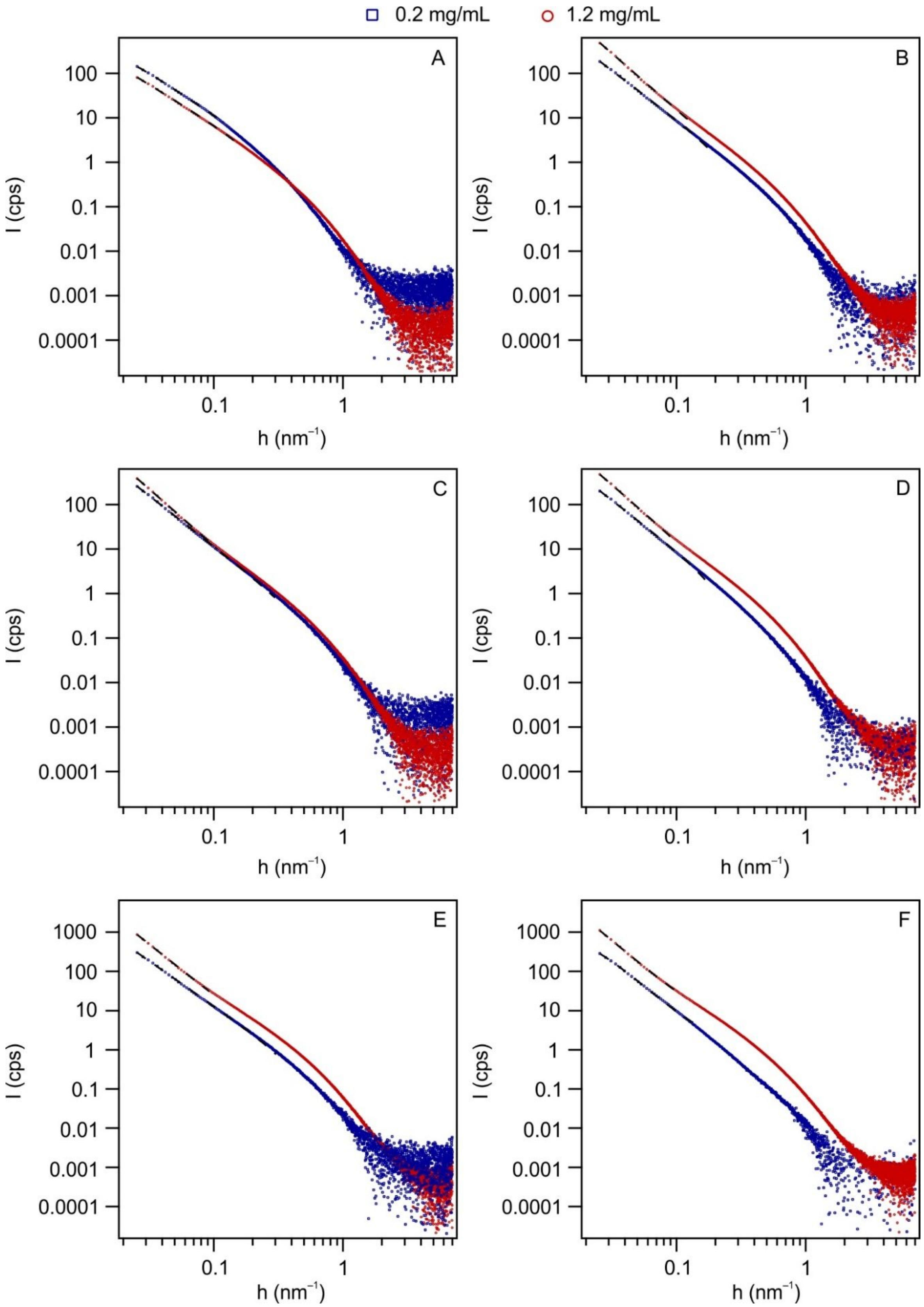

Figure S5. The SAXS scattering curves of dLDH (A), dLDHa (B), dLDHaH (C), dLDHaHt (D), dLDHaHtS (E), and dLDHaHtSC (F) at different particle concentrations.

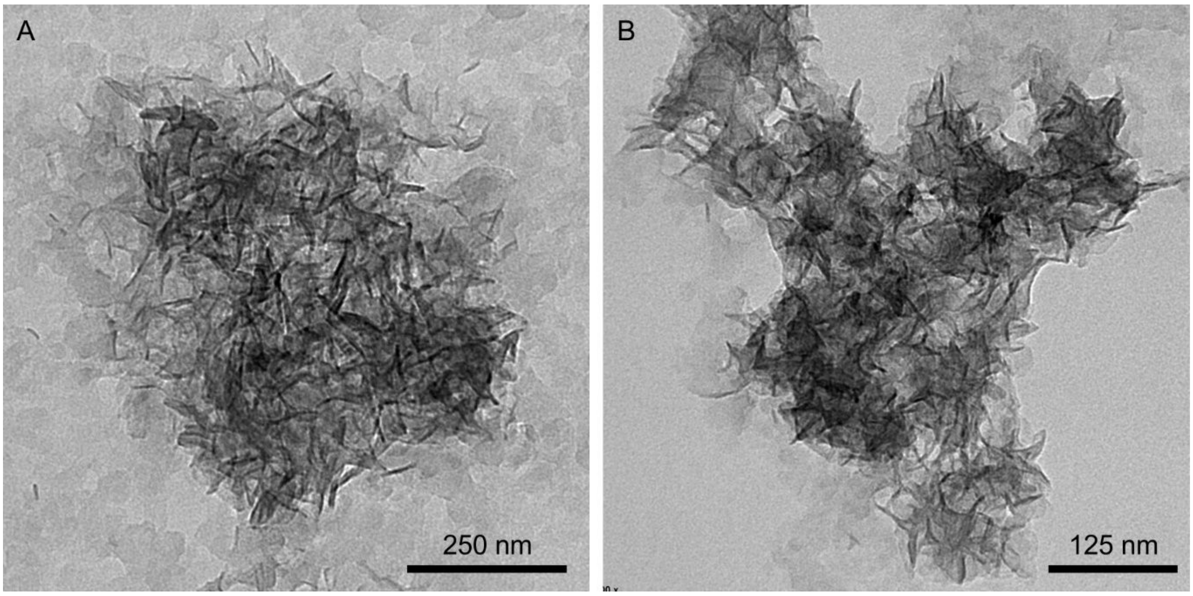

Figure S6. TEM micrographs of dLDH (A) and dLDHaHtSC (B) particles in solid state.

REFERENCES

1. Zhao, L.; Bashiri, S.; Toth, I.; Skwarczynski, M., Preparation of trimethyl chitosan-based polyelectrolyte complexes for peptide subunit vaccine delivery. In *Bacterial Vaccines: Methods and Protocols*, Bidmos, F.; Bossé, J.; Langford, P., Eds. Springer US: New York, NY, 2022; pp 141-149.
2. Zhang, Y. P.; Li, H. P.; Du, N.; Zhang, R. J.; Hou, W. G., Large-scale aqueous synthesis of layered double hydroxide single-layer nanosheets. *Colloid Surf. A-Physicochem. Eng. Asp.* **2016**, *501*, 49-54.
3. Delgado, A. V.; Gonzalez-Caballero, F.; Hunter, R. J.; Koopal, L. K.; Lyklema, J., Measurement and interpretation of electrokinetic phenomena. *J. Colloid Interface Sci.* **2007**, *309*, 194-224.
4. Russel, W. B.; Saville, D. A.; Schowalter, W. R., *Colloidal dispersions*. Cambridge University Press: Cambridge, 1989.
5. Hassan, P. A.; Rana, S.; Verma, G., Making sense of Brownian motion: Colloid characterization by dynamic light scattering. *Langmuir* **2015**, *31*, 3-12.
6. Trefalt, G.; Szilagyi, I.; Oncsik, T.; Sadeghpour, A.; Borkovec, M., Probing colloidal particle aggregation by light scattering. *Chimia* **2013**, *67*, 772-776.
7. Grolimund, D.; Elimelech, M.; Borkovec, M., Aggregation and deposition kinetics of mobile colloidal particles in natural porous media. *Colloid Surf. A* **2001**, *191*, 179-188.
8. Blanchet, C. E.; Spilotros, A.; Schwemmer, F.; Graewert, M. A.; Kikhney, A.; Jeffries, C. M.; Franke, D.; Mark, D.; Zengerle, R.; Cipriani, F.; Fiedler, S.; Roessle, M.; Svergun, D. I., Versatile sample environments and automation for biological solution X-ray scattering experiments at the P12 beamline (PETRA III, DESY). *J. Appl. Crystallogr.* **2015**, *48*, 431-443.
9. Li, Z. H.; Sun, J. H.; Wu, D.; Sun, Y. H.; Liu, Y.; Sheng, W. J.; Dong, B. Z., Determination of specific surfaces of silica xerogels by SAXS. *Chin. Sci. Bull.* **2000**, *45*, 1386-1390.
10. Li, Z. H.; Gong, Y. J.; Wu, D.; Sun, Y. H.; Wang, J.; Liu, Y.; Dong, B. Z., A negative deviation from Porod's law in SAXS of organo-MSU-X. *Microporous Mesoporous Mat.* **2001**, *46*, 75-80.
11. Beaucham, C.; Fridovich, I., Superoxide dismutase - improved assays and an assay applicable to acrylamide gels. *Anal. Biochem.* **1971**, *44*, 276-287.
12. Doerge, D. R.; Divi, R. L.; Churchwell, M. I., Identification of the colored guaiacol oxidation product produced by peroxidases. *Anal. Biochem.* **1997**, *250*, 10-17.
13. Johnson, K. A.; Goody, R. S., The original Michaelis constant: Translation of the 1913 Michaelis-Menten paper. *Biochemistry* **2011**, *50*, 8264-8269.

14. Hadwan, M. H.; Abed, H. N., Data supporting the spectrophotometric method for the estimation of catalase activity. *Data Brief* **2016**, *6*, 194-199.
15. Pavlovic, M.; Murath, S.; Katona, X.; Alsharif, N. B.; Rouster, P.; Maleth, J.; Szilagyi, I., Nanocomposite-based dual enzyme system for broad-spectrum scavenging of reactive oxygen species. *Sci. Rep.* **2021**, *11*, 4321.
16. Molnar, R.; Madacsy, T.; Varga, A.; Nemeth, M.; Katona, X.; Gorog, M.; Molnar, B.; Fanczal, J.; Rakonczay, Z.; Hegyi, P.; Pallagi, P.; Maleth, J., Mouse pancreatic ductal organoid culture as a relevant model to study exocrine pancreatic ion secretion. *Lab. Invest.* **2020**, *100*, 84-97.
17. Katana, B.; Rouster, P.; Varga, G.; Muráth, S.; Glinel, K.; Jonas, A. M.; Szilagyi, I., Self-assembly of protamine biomacromolecule on halloysite nanotubes for immobilization of superoxide dismutase enzyme. *ACS Appl. Bio Mater.* **2020**, *3*, 522-530.
18. Rouster, P.; Pavlovic, M.; Szilagyi, I., Immobilization of Superoxide Dismutase on polyelectrolyte functionalized titania nanosheets. *ChemBiochem* **2018**, *19*, 404-410.
19. Pavlovic, M.; Rouster, P.; Szilagyi, I., Synthesis and formulation of functional bionanomaterials with superoxide dismutase activity. *Nanoscale* **2017**, *9*, 369-379.
20. Pavlovic, M.; Rouster, P.; Somosi, Z.; Szilagyi, I., Horseradish peroxidase-nanoclay hybrid particles of high functional and colloidal stability. *J. Colloid Interface Sci.* **2018**, *524*, 114-121.
21. Rouster, P.; Pavlovic, M.; Saringer, S.; Szilagyi, I., Functionalized titania nanosheet dispersions of peroxidase activity. *J. Phys. Chem. C* **2018**, *122*, 11455-11463.
22. Sáringer, S.; Valtner, T.; Varga, Á.; Maléth, J.; Szilagyi, I., Development of polymer-based multifunctional composite particles of protease and peroxidase activities. *J. Mat. Chem. B* **2022**, *10*, 2523-2533.
23. Saringer, S.; Rouster, P.; Szilagyi, I., Co-immobilization of antioxidant enzymes on titania nanosheets for reduction of oxidative stress in colloid systems. *J. Colloid Interface Sci.* **2021**, *590*, 28-37.
24. Zhang, S. H.; Jiang, Z. Y.; Zhanga, W. Y.; Wang, X. L.; Shi, J. F., Polymer-inorganic microcapsules fabricated by combining biomimetic adhesion and bioinspired mineralization and their use for catalase immobilization. *Biochem. Eng. J.* **2015**, *93*, 281-288.
25. Chang, F. P.; Chen, Y. P.; Mou, C. Y., Intracellular implantation of enzymes in hollow silica nanospheres for protein therapy: Cascade system of Superoxide Dismutase and Catalase. *Small* **2014**, *10*, 4785-4795.
